# Research Progress on the Mechanism of Cochlear Hair Cell Regeneration

**DOI:** 10.3389/fncel.2021.732507

**Published:** 2021-08-20

**Authors:** Shan Xu, Ning Yang

**Affiliations:** Department of Otolaryngology, The First Hospital of China Medical University, Shenyang, China

**Keywords:** inner ear, stem cell, hair cell, regeneration, sensorineural hearing loss

## Abstract

Mammalian inner ear hair cells do not have the ability to spontaneously regenerate, so their irreversible damage is the main cause of sensorineural hearing loss. The damage and loss of hair cells are mainly caused by factors such as aging, infection, genetic factors, hypoxia, autoimmune diseases, ototoxic drugs, or noise exposure. In recent years, research on the regeneration and functional recovery of mammalian auditory hair cells has attracted more and more attention in the field of auditory research. How to regenerate and protect hair cells or auditory neurons through biological methods and rebuild auditory circuits and functions are key scientific issues that need to be resolved in this field. This review mainly summarizes and discusses the recent research progress in gene therapy and molecular mechanisms related to hair cell regeneration in the field of sensorineural hearing loss.

## Introduction

Neural stem cells have the ability to self-renew and differentiate into various types of nerve cells and have been used as a potential treatment for various diseases. Some supporting cells with proliferation ability in the inner ear are also called sensory precursor cells (Monzack and Cunningham, 2013). Stem cell therapy refers to a treatment that uses the diversity of stem cells to induce differentiation into the same structure as the original shape and function when the normal structure of the organism is damaged or changed. This treatment can supplement the defective and damaged inner ear hair cells. The differentiation mechanism of inner ear stem cells is a complex regulatory network system. In addition to the expression sequence of genes playing an important role in the regulation of their differentiation, the secretion and interaction of various cytokines are also closely related to it. At present, the molecular regulation mechanism of inner ear stem cells is still unclear. With the increase of related research and the development of molecular biology technology, the molecular regulation mechanism of neural stem cell differentiation has gradually been elucidated. In recent years, the inner ear sensory precursor cells or stem cells are induced to re-enter the cell cycle by activating the inner ear-related signal pathways, and proliferate and differentiate into hair cells, and finally restore hearing or vestibular function, which have gradually become a research hotspot (Monzack and Cunningham, 2013). The proliferation and differentiation of sensory precursor cells are regulated by various related signal pathways, including WNT, Notch, BMP/Smad, FGF, IGF, and Shh signal pathways (Schimmang, 2007; Munnamalai and Fekete, 2016; Waqas et al., 2016; Wu et al., 2016). The regulation of these signal pathways and related factors is very important for the induction of inner ear stem cells and sensory precursor cells to differentiate into mature inner ear hair cells.

In recent years, with the in-depth research on the pathogenesis of deafness, more and more researchers are trying to treat hearing diseases through stem cell therapy. At present, there are two main ways to regenerate hair cells in the inner ear: one is that the supporting cells will re-enter the cell cycle and differentiate into hair cells after mitosis. Another way is that supporting cells will directly transdifferentiate into hair cells. In addition to Sox2^+^ cells, Lgr5^+^ cells, and Axin2^+^ cells that are often used for stem cell research, Chai et al. (2012) found that the cell population of Fzd9^+^ cells is much smaller than Lgr5^+^ progenitor cells in the cochlea of newborn Fzd9-CreER/Rosa26-tdTomato mice (Zhang et al., 2019). They also found that Fzd9^+^ cells have the ability to generate hair cells *in vivo*. In addition, the proliferation, differentiation, and hair cell generation capabilities of Fzd9^+^ cells cultured *in vitro* are similar to those of Lgr5^+^ progenitor cells. Therefore, Fzd9^+^ cells may be the main functional progenitor cell type in Lgr5^+^ cells (Zhang et al., 2019). Therefore, Fzd9 can be used as a marker for hair cell progenitor, which is more specific than Lgr5 in hair cell progenitor.

## Application of AAV Vectors in Inner Ear

The method of regulating the differentiation and development of stem cells through gene editing to promote the regeneration of inner ear hair cells has gradually become a research hotspot in the field of hearing. The ideal vector for gene editing needs to accurately deliver the nucleic acid fragments into the inner ear and target cells and express them efficiently. In addition, the vector needs to have high transfection efficiency, controllable intensity and time, and high safety. Traditional viral vectors are toxic and carry limited gene capacity. In recent years, researchers have discovered some ideal adeno-associated virus (AAV) vectors that can be transfected in the inner ear (Iizuka et al., 2008; Giannelli et al., 2018; Gu et al., 2019; Tan et al., 2019). AAV vector-mediated gene therapy has been approved in the United States and can be used to treat rare hereditary eye diseases (Tan et al., 2019). Omar Akil et al. have used the AAV virus vector to deliver the *VGLUT3* gene to VGLUT3 knockout mice and achieved significant improvement in hearing (Akil et al., 2012). In order to avoid the shortcomings of low efficiency of traditional AAVs infecting outer hair cells, Zinn et al. made the vector AAV2/Anc80L65 based on the original sequence of the AAV capsid (Zinn et al., 2015). This vector can efficiently target the inner hair cells and outer hair cells of the cochlea, which indicates an important breakthrough in the research of AAV vectors in cochlear cell targeting. Kevin and colleagues found that AAV2.7m8 can also efficiently infect inner hair cells and outer hair cells in the cochlea, and the efficiency of infecting outer hair cells is even higher than that of Anc80L65 (Gu et al., 2019; Isgrig et al., 2019). In addition, AAV2.7m8 not only preferentially targets cochlear hair cells, but it can also efficiently infect Lgr5^+^ supporting cells. Tan et al. constructed the AAV mutant AAV-inner ear (AAV-ie) by inserting the polypeptide DGTLAVPFK, which can increase the infection efficiency by generating a transmembrane structure (Tan et al., 2019). AAV-ie can efficiently infect cochlear hair cells and vestibular hair cells. In the cochlea, since GFAP protein is expressed in supporting cells but not in hair cells, the GFAP promoter can be used to achieve specific expression of AAV-ie genes in supporting cells. In addition, studies have found that AAV-ie does not affect the function of hair cells and the auditory system (Tan et al., 2019).

## Signal Pathways Related to Hair Cell Regeneration

**Figure 1:**
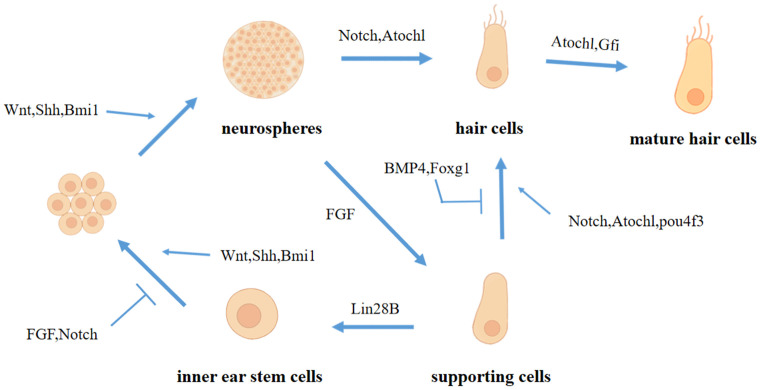
The role of various signal pathways and related factors in the process of hair cell regeneration.

In recent years, researchers have discovered through the study of related pathways in the inner ear that WNTs, FGFs, BMP, Shh, Notch, and other signaling molecules play different roles in the process of hair cell regeneration (Figure 1). Therefore, hair cell regeneration does not depend on the activation of one signal pathway but is coordinated by multiple signal pathways.

### The Role of Wnt Signaling Pathway in Hair Cell Regeneration

The WNT/β-catenin signaling pathway plays an important regulatory role in cell proliferation, cell fate determination, and hair cell differentiation (Jacques et al., 2014). When the WNT/β-catenin signal is inhibited, the proliferation ability of sensory cells is reduced, and the activation of the WNT/β-catenin signal promotes the increase of hair cells (Jacques et al., 2012; Shi et al., 2014). The target genes *Lgr5* and *Lgr6* of the WNT pathway are expressed in embryonic and neonatal cochlear progenitor cells (Chai et al., 2011; Samarajeewa et al., 2019). Lgr5^+^ cells have the characteristics of hair cell progenitor cells, such as proliferation, self-renewal, and regeneration into hair cells (Chai et al., 2012; Shi et al., 2012; Cox et al., 2014; Wang et al., 2015). After WNT agonist treatment, the proliferation ability of the Lgr5^+^ progenitor cells of the neonatal mouse cochlea and their ability to differentiate into hair cells were enhanced (Romero-Carvajal et al., 2015; Samarajeewa et al., 2019). WNT agonists or overexpression of β-catenin can increase the proliferation of Lgr5^+^ progenitor cells and promote the formation of hair cells, while WNT antagonists inhibit the proliferation of Lgr5^+^ cells and the ability to regenerate hair cells (Chai et al., 2012; Shi et al., 2012, 2013). In newborn mice, the activation of WNT signaling can also cause Axin2-positive cells to proliferate and differentiate into hair cells and supporting cells (Jan et al., 2013). When the expression of β-catenin and Atoh1 are activated simultaneously, the proliferation ability and the directed differentiation ability of Lgr5^+^ cells into hair cells and the survival ability of newly regenerated hair cells are enhanced (Kuo et al., 2015; Mittal et al., 2017). In addition, the combination of WNT signal activation and Notch signal inhibition in the cochlea of newborn mice has also been shown to promote the proliferation of supporting cells and the regeneration of hair cells (Ni et al., 2016).

### The Role of Notch Signaling Pathway in Hair Cell Regeneration

The Notch signaling pathway plays an important role in the development and regeneration of hair cells. Supporting cells are induced to transdifferentiate into hair cells when Notch signaling in the cochlea of newborn mice is suppressed (Yamamoto et al., 2006; Mizutari et al., 2013; Bramhall et al., 2014). Researchers found that activating the Notch pathway in supporting cells puts them in a quiescent state and inhibits hair cell regeneration (Ma et al., 2008; Daudet et al., 2009). Previous studies found that in the cochlea of neonatal mammals, the expression of Atoh1 was up-regulated after γ-secretase inhibitor blocked the Notch signal, and caused the adjacent supporting cells to transdifferentiate into hair cells. However, this newly generated hair cell does not have the characteristics of a mature hair cell (Korrapati et al., 2013; Mizutari et al., 2013). In addition, knocking out Hes1 and Hes5 members of the Hes family with siRNA can also up-regulate the expression of Atoh1, leading to an increase in the number of hair cells in the cochlea of newborn and adult mice (Du et al., 2018). Conditional inhibition of Notch signal can accelerate Lgr5^+^ cells in supporting cells to become new hair cells (Mittal et al., 2017). Co-activating the cell cycle activator Myc and Notch1 in the inner ear can induce the proliferation of different types of cochlear sensory epithelial cells. After the cochlear supporting cells are reprogrammed by regulating the activity of MYC/NICD, Atoh1 can induce the supporting cells *in vivo* and *in vitro* to effectively transdifferentiate into hair cell-like cells (Shu et al., 2019). Li et al. (2015) found that the combined inhibition of WNT and Notch signals can reduce the generation of hair cells, indicating that the progenitor cell proliferation phenomenon that occurs after Notch inhibition is mainly regulated by the WNT pathway (Li et al., 2015).

### The Role of Hedgehog Signaling Pathway in Hair Cell Regeneration

The Hedgehog pathway is a highly conserved signaling pathway, which plays an important role in regulating the proliferation and cell fate determination and differentiation of progenitor cells in the early developmental stage of the inner ear (Zarei et al., 2017). Previous studies have found that inactivation of Hedgehog signaling causes abnormal proliferation and differentiation of mammalian cochlear cells, leading to abnormal development of cochlear structure and function (Brown and Epstein, 2011; Bok et al., 2013; Son et al., 2015). Lu et al. (2013) found that after neomycin damage, Shh signaling may inhibit the function of pRb to promote sensory epithelial cells in the cochlea of newborn mice to re-enter the cell cycle and regenerate into new hair cells. Chen et al. (2017) found that recombinant Shh protein can effectively promote the proliferation and differentiation of Lgr5^+^ progenitor cells in the cochlea of newborn mice. In addition, after R26-SmoM2 mouse cochlear explants are treated with neomycin, Hedgehog signal activation can significantly promote cochlear epithelial cell proliferation and hair cell regeneration (Chen et al., 2017).

### The Role of FGF Signaling Pathway in Hair Cell Regeneration

The FGF signaling pathway activates the gene regulatory network of the early development of the inner ear and induces the formation of the pre-placodal region and the auditory placode (Padanad et al., 2012; Ornitz and Itoh, 2015). In addition, the FGF signaling pathway plays a key role in the spontaneous regeneration of lower vertebrate hair cells and the induction and differentiation of stem cells into hair cells. Ku et al. found that when the chicken utricle was damaged by ototoxic drugs, the decrease of FGF expression level promoted the proliferation of supporting cells (Ku et al., 2014). Jiang et al. found that the inhibition of the FGF and Notch pathway in the process of supporting cell proliferation is consistent with the down-regulation of the cycl-dependent protein kinase (CDK) inhibitor CDKN1b (p27/kip1; Jiang et al., 2014). Therefore, the inhibition of FGF and Notch signaling pathway is the triggering factor for supporting cell proliferation. Piotrowski et al. found through scRNA-seq that the deletion of *Fgf3* expression in zebrafish supporting cells resulted in enhanced hair cell regeneration and increased the number of cells in neuromast (Lush et al., 2019). Lee et al. (2016) used drugs and genes to block FGF signaling and found that inhibiting FGF signaling can significantly inhibit the regeneration of hair cells in neuromast. Tang et al. found that the FGF signaling pathway and WNT signaling pathway have a coordinated regulatory effect on the proliferation of zebrafish lateral line cells (Tang et al., 2019). When the FGF signal is inhibited, the WNT pathway-mediated cell proliferation will also be inhibited. In the absence of WNT activity, activating the FGF signal with bFGF will restore part of supporting cell proliferation and hair cell regeneration.

## Transcription Factors Related to Hair Cell Regeneration

Atoh1 is a transcription factor with a helix-loop-helix structure, which is essential for the differentiation of hair cells during the development of the inner ear (Bermingham et al., 1999). In animal experiments, it was found that the sensory epithelium of the cochlea of Atoh1 knockout mice differentiated into supporting cells, but not cochlear hair cells and vestibular hair cells (Chen et al., 2002). Therefore, Atoh1 gene is an essential transcription factor in the process of hair cell generation. In *in vitro* experiments, researchers found that activating the expression of Atoh1 in the cochlea or vestibular sensory epithelium can induce hair cell regeneration (Bermingham et al., 1999; Izumikawa et al., 2005; Gubbels et al., 2008; Liu et al., 2012). In damaged mouse utricle, overexpression of Atoh1 can induce the proliferation of supporting cells and promote them transdifferentiate into hair cell-like cells (Jen et al., 2019). In addition, the overexpression of Atoh1 also caused the up-regulation of many hair cell-related genes but did not include the hair cell maturation-related genes. Cheng found that the expression of Atoh1 in prenatal rats can induce supporting cells to differentiate into sensory hair cells, but the regenerated hair cells are immature, which may be due to the absence of Atoh1 down-regulation during hair cell development (Cheng, 2019). Therefore, hair cell regeneration and maturation are not only regulated by Atoh1, but also coordinated by other transcription factors.

Gfi1 is one of the GPS (Gfi1/pag3/SENS) family transcription factors with a zinc finger domain. During the development of the inner ear, Gfi1 plays an important role in the normal differentiation, survival, and maturation of hair cells (Wallis et al., 2003). Gfi1 expression-deficient mice showed abnormal hair cell development, such as cochlear outer hair cells and inner hair cells degenerating from the basal turn to the apex turn until they were completely absent (Wallis et al., 2003; Li and Doetzlhofer, 2020). Matern et al. found that when Gfi1 expression is absent, the maturation of hair cells in the cochlea and vestibule is blocked, and the expression of hair cell maturation markers, such as Strc, Tmc1, and Ocm, is inhibited (Matern et al., 2020). Lee et al. found that Gfi1 and upstream Atoh1 coordinately regulate the regeneration of cochlear hair cells in newborn mice (Lee et al., 2020).

The secreted protein bone morphogenetic protein 4 (BMP4) can regulate inner ear morphogenesis and hair cell development. Lewis et al. found that when the cochlea cultured *in vitro* is damaged, the addition of BMP4 can inhibit the expression of Atoh1 in supporting cells, thereby inhibiting supporting cell mitosis and hair cell regeneration (Lewis et al., 2018). After treatment with the BMP4 inhibitor noggin, the number of regenerated hair cells increased (Lewis et al., 2018).

Bmi1 is a member of the Polycomb protein family and plays a regulatory role in the proliferation of progenitor cells and stem cells in multiple organs (Bruggeman et al., 2007; López-Arribillaga et al., 2015; Lu et al., 2017). Recently, researchers have discovered that Bmi1 activates WNT signaling by inhibiting the Dickkopf (DKK) family with WNT inhibitor functions (Cho et al., 2013). Lu et al. (2017) demonstrated in *in vitro* experiments that knocking out Bmi1 can significantly inhibit the proliferation of Lgr5^+^ progenitor cells in the cochlea of neonatal mice after neomycin damage. Compared with wild-type mice, the expression of DKK1 in Bmi1^−/−^ neonatal mice was significantly up-regulated, while the expression of β-catenin and Lgr5 was significantly down-regulated. In addition, the WNT agonist BIO inhibited the decline of the proliferation ability of Bmi1^−/−^ mouse supporting cells, indicating that Bmi1 affects the proliferation of supporting cells and Lgr5^+^ progenitor cells through the regulation of WNT signaling pathway. Therefore, Bmi1 may be a new therapeutic target for hair cell regeneration.

The POU domain transcription factor Pou4f3 plays an important role in the development of inner ear hair cells (Masuda et al., 2011). Recent studies have found that in the adult mouse cochlea, the ectopic expression of Pou4f3 can promote the transdifferentiation of supporting cells into hair cells together with Atoh1 (Walters et al., 2017). The activation of downstream target genes by Pou4f3 can promote Atoh1-mediated hair cell development and survival (Walters et al., 2017). Pou4f3 not only regulates the expression of Gfi1 and Nr2f2 related to hair cell regeneration (Zhong et al., 2019) but also is the direct target gene of Atoh1 during hair cell development (Lee et al., 2020). Atoh1-Pou4f3-target gene (such as Gfi1) is not only an important molecular pathway for regulating the fate and differentiation of hair cells but also an important molecular pathway for hair cell maturation and survival. Therefore, POU4f3 as a therapeutic target, activating its activity alone or coordinating with Atoh1 may promote the regeneration of auditory hair cells.

Foxg1 is one of the forkhead box genes involved in morphogenesis, cell fate determination, and proliferation. Previous studies reported that Foxg1 is necessary for the morphogenesis of the inner ear in mammals (Pauley et al., 2006; Hwang et al., 2009; He et al., 2018). Pauley et al. found that in Foxg1 knockout mice, the cochlear duct became shorter, the number of hair cells increased, the vestibule shrank, the growth of axons, and the distribution of vestibular neurons were abnormal (Pauley et al., 2006). Chai et al. (2012) found that conditionally knocking out *Foxg1* in Sox2^+^ supporting cells and Lgr5^+^ progenitor cells of newborn mice induced these cells to transdifferentiate into hair cells, resulting in a significant increase in the number of hair cells and a significant decrease in supporting cells (Zhang et al., 2020). In addition, they also found that knocking out Foxg1 expression down-regulated the expression of several Notch signaling pathway factors such as Hes1, Hes5, and Hey1. At the same time, the expression of cell cycle-dependent kinase (Cdk2) was also down-regulated, while the expression of cell cycle repressors Cdkn1c, Cdkn2a, and Gadd45g were up-regulated, indicating that Foxg1 knockout may cause cell cycle and Notch signaling pathway to be inhibited, which led to the increase of hair cells (Zhang et al., 2020). Chai et al. (2012) also found that knocking out Foxg1 in Sox9^+^ supporting cells can promote the transdifferentiation of supporting cells into hair cells in neonatal mouse utricle (Zhang et al., 2020). The above studies provide evidence for the role of Foxg1 in the regeneration of cochlear hair cells in newborn mice.

The Lin28 gene encodes an evolutionarily highly conserved RNA binding protein, which is known to regulate the larval development time of Caenorhabditis elegans (Moss and Tang, 2003). In humans and mice, Lin28a and its homolog Lin28b are key regulators of organ growth, metabolism, tumorigenesis, and tissue repair (Ambros and Horvitz, 1984; Shyh-Chang and Daley, 2013). Doetzlhofer and colleagues found that the pathway composed of *Lin28b* and *let-7* miRNAs regulates the generation of new hair cells in P2 mouse cochlear explants (Golden et al., 2015). The role of *Lin28b* is mainly to promote the generation of new hair cells, while the role of *let-7* miRNAs is to inhibit the generation of new hair cells. *Lin28b* functional defect or overexpression of *let-7g* miRNAs leads to the inhibition of the activity of Akt-mTOR complex 1 (mTORC1) so that immature supporting cells cannot be transdifferentiated into hair cells (Li and Doetzlhofer, 2020). On the contrary, overexpression of Lin28b increased the activity of Akt-mTORC1, and dedifferentiated the maturing supporting cells into progenitor-like cells, and generated hair cells through mitotic and non-mitotic mechanisms (Li and Doetzlhofer, 2020). These findings may provide new strategies for future hair cell regeneration treatments.

In addition, Menendez et al. (2020) found that the combination of four transcription factors (Six1, Atoh1, Pou4f3, and Gfi1) can transdifferentiate neonatal mouse supporting cells (P8), mouse embryonic fibroblasts, and adult mouse tail fibroblasts into induced hair cells (IHCs). IHCs have various characteristics of hair cells, such as morphology, transcriptome and epigenetic characteristics, electrophysiological characteristics, mechanosensory channel expression, and ototoxin susceptibility. Therefore, IHCs can be used as an ideal *in vitro* model for studying hair cell function, maturation, regeneration, and ototoxin sensitivity.

## The Role of Epigenetic Regulation of Hair Cell Regeneration

Epigenetic modification plays an important role in the development of the inner ear, and recent studies have found that it also has an important regulatory role in hair cell regeneration. During the development of zebrafish larvae, the inhibition of LSD1 by 2-PCPA decreased the expression of WNT/β-catenin and FGF signaling pathways, thereby significantly inhibiting the regeneration of supporting cells and hair cells after neomycin damage (He et al., 2016). The inhibition of G9a/GLP by BIX01294 significantly reduced the dimethylation of H3K9 in the zebrafish lateral line (Tang et al., 2016). The defect of H3K9me2 significantly inhibited the WNT/β-catenin and FGF signaling pathways, which significantly reduced the proliferation of supporting cells after neomycin damage, and ultimately lead to the reduction of mitotic regeneration of hair cells in the zebrafish lateral line (Tang et al., 2016). Previous studies have found that when 5-azacytidine, a DNA methyltransferase (Dnmt) inhibitor, is injected into the cochlea of mature mice that are chemically deafened, DNA demethylation may promote the regeneration of hair cells in the cochlea of mature mice (Deng and Hu, 2020). The advantage of this epigenetic method is that the DNA sequence remains unchanged during the process without integrating the exogenous DNA sequence.

## Conclusion

There are various ways and mechanisms that cause sensorineural hearing loss, among which irreversible damage to inner ear hair cells is the main cause of sensorineural hearing loss. Although the commonly used hearing aids and cochlear implants in clinical practice also improve the hearing of patients, their effect depends on the quantity and quality of residual hair cells and spiral neurons. Therefore, the ideal way to treat sensorineural hearing loss is to regenerate hair cells through stem cells to repair the structure and function of the cochlea so as to fundamentally restore hearing. Stem cell therapy in the auditory field has been a research hotspot in recent years. Although some progress has been made, almost all are results at the animal level, and there is still a long way to go before clinical transformation. The microenvironment of inner ear stem cells and the interaction with neighboring cells are very important for inner ear stem cells or sensory precursor cells to induce differentiation into mature inner ear hair cells. In the reported studies, the efficiency of differentiation of inner ear stem cells or sensory precursor cells into hair cells is still low. An insufficient number of new hair cells, immature new hair cells without the function of mature hair cells, and long–term survival of new hair cells are all key problems and difficulties that need to be solved urgently. All these indicate that it is more difficult to regulate a single signal pathway to regenerate functional hair cells, and it may require coordinated regulation of multiple genes to effectively promote hair cell regeneration and the functional maturity and survival of new hair cells. At present, inducing the committed differentiation of stem cells into hair cells or nerve cells, the exploration of the methods of stem cell transplantation into the inner ear, and the safety research of stem cell transplantation have laid the foundation for the transplantation of stem cells *in vivo*.

## Author Contributions

SX and NY wrote the article. All authors contributed to the article and approved the submitted version.

## Conflict of Interest

The authors declare that the research was conducted in the absence of any commercial or financial relationships that could be construed as a potential conflict of interest.

## Publisher’s Note

All claims expressed in this article are solely those of the authors and do not necessarily represent those of their affiliated organizations, or those of the publisher, the editors and the reviewers. Any product that may be evaluated in this article, or claim that may be made by its manufacturer, is not guaranteed or endorsed by the publisher.

## Abbreviations

AAV: adeno-associated virus
AAV-ie: AAV-inner ear
CDK: cycl-dependent protein kinase
BMP4: bone morphogenetic protein 4
DKK: Dickkopf
mTORC1: Akt-mTOR complex 1
Dnmt: DNA methyltransferase.
